# Antipsychotic prescribing of consultant forensic psychiatrists working in different levels of secure care with patients with schizophrenia

**DOI:** 10.1192/pb.bp.115.053009

**Published:** 2017-04

**Authors:** Anna Machin, Lucy McCarthy

**Affiliations:** 1 East Midlands Training Scheme, Nottingham; 2 East Midlands Centre for Forensic Mental Health, Leicester

## Abstract

**Aims and method** To detect any differences in the antipsychotic prescribing practices of consultant forensic psychiatrists working in different levels of secure care with patients diagnosed with schizophrenia, and to identify potential reasons for any differences. Prescribing data were collected from four secure hospitals within one National Health Service trust. A questionnaire was sent to consultant forensic psychiatrists working at those hospitals as well as those working in the trust's community forensic services.

**Results** Consultants working in high security prescribed more oral antipsychotics than consultants working in medium and low security, who prescribed more depot antipsychotics, as established via the prescribing data. The questionnaire provided insight regarding the reasons for these preferences.

**Clinical implications** There were differences in the antipsychotic prescribing practices of consultant forensic psychiatrists working in different levels of secure care, and, overall, the rate of depot antipsychotic prescribing was lower than might be expected. Although it was positive that the rate of polypharmacy was low when compared with earlier studies, the lower-than-expected rate of depot antipsychotic prescribing has clinical implications.

Schizophrenia is an enduring mental illness characterised by remissions and relapses. Treatment is available in the form of psychosocial interventions but the mainstay of effective management is antipsychotic medication.^1–3^ Antipsychotic efficacy is reduced by poor adherence and it is estimated that the risk of relapse is 2 to 6 times greater for patients not taking antipsychotic treatment.^1^

Forensic psychiatrists working in secure hospitals are likely to have case-loads with high levels of comorbidity, non-adherence and risk;^4,5^ a history of aggression has been found to predict the use of high-dose antipsychotic treatment (total antipsychotic doses totalling greater than 100% maximum *British National Formulary* dose).^1^

Paton *et al*^6^ reported on an in-patient census. Most (97%) of the 53.2% diagnosed with psychosis were prescribed an antipsychotic. Polypharmacy (the prescription of more than one antipsychotic agent) was common. Of those prescribed a regular antipsychotic, 51.6% were prescribed a first-generation agent, 11.3% were prescribed clozapine and 33% were prescribed a depot agent (80% of these patients had a psychotic illness). High-dose prescribing was more common when a depot agent was prescribed.

No previous studies have examined the prescribing patterns of forensic psychiatrists working in differing levels of security. It is important to identify such patterns to build on existing knowledge and to identify areas requiring change.

## Aims

To detect any differences in the antipsychotic prescribing practices of consultant forensic psychiatrists working in different levels of secure care within a National Health Service (NHS) trust, and to identify potential reasons for any differences. We hypothesised that there would be no significant differences in prescribing patterns in this clinician group.

## Method

### Settings and sample

Covering a vast geographical area, the NHS trust studied is one of the largest in England and provides services in a variety of settings. Its secure hospitals feature one high secure hospital, two medium secure hospitals and one low secure hospital. Information on antipsychotic prescribing was collected on all in-patients with schizophrenia in these four hospitals in July 2014. A questionnaire was sent to the 34 consultant forensic psychiatrists based at the hospitals and 3 community-based consultant forensic psychiatrists working within the same trust.

### Procedure

#### Prescribing patterns

In July 2014, the electronic healthcare records were used to produce a list of all current in-patients at the trust's four secure hospitals. The clinical records (electronic and paper healthcare records, and pharmacy records) were then used to ascertain which of these patients had schizophrenia. Further information was collected about patients with schizophrenia (gender, age, ethnicity, length of stay, antipsychotic medication prescribed), to produce an anonymised data-set detailing the percentage of patients diagnosed with schizophrenia at each hospital, and the proportion of patients with schizophrenia prescribed different types of antipsychotic medication.

#### Consultant questionnaires

Owing to the absence of a validated instrument, a semi-structured self-report questionnaire was developed to assess forensic consultants' attitudes to the prescription of oral and depot antipsychotic medications. The questionnaire was designed to take less than 5 min to complete. Consultants were asked in which level of security they worked and then three further questions:

1. Disregarding the special case of clozapine, when treating a patient with schizophrenia do you have a general preference for either oral or depot antipsychotic medications? (Yes/No)
2. Please indicate the strength of any preference on the scale below (0–100 anchored Likert scale: 0 – oral, 100 – depot).
3. What are the reasons behind any stated preference? ((a) Improves adherence, (b) Better clinical outcome, (c) More convenient for the patient, (d) More convenient for the clinical team, (d) The next level of security/community team would expect/prefer it).

There was also space for a free-text response. The three community consultants were asked two further questions:

1. How important is the route of antipsychotic administration in your considering whether to accept a patient on to your caseload? (0–100 anchored Likert scale: 0 – very important, 100 – not at all important).
2. How likely are you to accept a patient currently prescribed an oral antipsychotic (not clozapine)? (0–100 anchored Likert scale: 0 – very unlikely, 100 – very likely).

The Likert scale is an ordinal psychometric assessment of attitudes or opinions, typically lacking concrete answers to accommodate neutral or undecided feelings. It was selected for this questionnaire owing to the speed and ease of completion, low cost, ease of distribution, and providing results amenable to analysis.

The Likert scale has been criticised for failing to measure the true attitudes of respondents, as it gives only 5 to 7 options of choice, and also for the space between each choice in reality possibly not being equidistant. In view of this, and in an attempt to further maximise freedom on behalf of the responder and to avoid railroading respondents into giving polarised responses, the Likert scale was amended to include some characteristics of an analogue scale.

The questionnaire was sent, with a cover letter, to all forensic consultants working in the four secure hospitals and in community forensic services in the NHS trust. After 3 months, the questionnaire was sent again to encourage non-responders. All data were supplied anonymously.

### Ethical considerations

The project proposal was reviewed by the trust's research and innovations department and approved as service evaluation; research ethics approval was therefore not required. Patient anonymity was preserved throughout the study.

### Analytic strategy

SPSS version 21 (Windows 10) was used for data analysis. Chi-square and ANOVA were used where appropriate. All tests were two-tailed and *P* ⩽ 0.05 was used to determine statistical significance.

## Results

### Demographic information

In July 2014, there were 556 patients detained at the four secure hospitals; 265 (48%) were diagnosed with schizophrenia. The sample characteristics are summarised in Table 1.

**Table 1:** Sample characteristics of in-patients at each of the four secure hospitals

	High secure hospital	Medium secure hospital 1	Medium secure hospital 2	Low secure hospital	*P*^a^	
Total number	339	69	85	63	–	

Schizophrenia, *n* (%)	139 (41)	53 (77)	33 (39)	40 (63)	**0.001**	χ^2^ = 38.52, d.f. = 3

Ethnicity, *n* (%)						
White British	100 (72)	41 (77)	21 (64)	17 (42)	**0.002**	χ^2^ = 15.23, d.f. = 3
White other	5 (4)	0 (0)	1 (3)	2 (5)	0.500	χ^2^ = 2.34, d.f. = 3
Black/Black British	19 (14)	3 (6)	4 (12)	11 (28)	**0.026**	χ^2^ = 9.25, d.f. = 3
Asian/Asian British	9 (6)	5 (9)	2 (6)	3 (7)	0.902	χ^2^ = 0.58, d.f. = 3
Mixed/other ethnicity	6 (4)	4 (8)	5 (15)	7 (18)	**0.024**	χ^2^ = 9.42, d.f. = 3

Age, years						
Mean (s.d.)	40.0 (9.8)	36.9 (8.7)	36.5 (8.8)	38.1 (10.3)	0.100	ANOVA *F*_(3,261)_ = 2.10
Median (range)	38.6 (21.9–66.3)	37.2 (19.6–60.0)	35.2 (21.8–58.2)	35.9 (19.8–62.0)	–	

Length of stay, years						
Mean (s.d.)	6.4 (4.5)	2.1 (1.9)	1.9 (1.4)	3.0 (3.9)	**0.001**	ANOVA *F*_(3,261)_ = 26.86
Median (range)	5.9 (0.1–21.4)	1.5 (0.1–8.0)	1.4 (0.03–5.6)	1.5 (0.03–18.2)	–	

Female patients, *n* (%)^b^	5 (3.6)	0 (0)	1 (3.0)	4 (10.0)	0.093	χ^2^ = 0.58, d.f. = 3
Age, years: mean (s.d.)	43.1 (5.9)	–	–	41.7 (13.4)	0.373	ANOVA *F*_(2,7)_ = 1.14
Age, years: median (range)	41.1 (36.9–54.3)	–	–	39.7 (25.7–62.0)	–	

Length of stay, years: mean (s.d.)	4.9 (2.4)	–	–	1.1 (0.4)	0.053	ANOVA *F*_(2,7)_ = 4.62
Length of stay, years: median (range)	6.5 (1.3–6.8)	–	–	1.1 (0.5–1.6)	–	

a. Bold denotes significance (*P* ⩽ 0.05).

b. As medium secure hospital 2 had only 1 female in-patient, means and medians for age and length of stay have not been calculated.

Medium secure hospital 1 provides a male-only service. The high secure hospital and medium secure hospital 2 have wards specialising in the care of patients with personality disorder, whereas medium secure hospital 1 and the low secure hospital do not, hence the differences in the rate of schizophrenia. The proportion of patients from Black and minority ethnic (BME) groups was high when compared with the general population^7^ (29% *v.* 14% respectively). The rate was highest for the low secure hospital (53%). This significant finding mirrors an earlier study^8^ which found an overrepresentation of BME groups admitted to low secure services across the UK.

There was little difference in mean patient age between the four hospitals, and expected differences in the mean lengths of stay.

### Pattern of prescribing

Of all patients with schizophrenia, 3% (*n* = 8) were not prescribed antipsychotic medication and 12% (*n* = 33) were prescribed antipsychotic medication constituting polypharmacy. The most common polypharmacological combination was clozapine augmented with a second-generation oral antipsychotic. Data regarding the prescription of antipsychotic medication are summarised in Table 2.

**Table 2:** Antipsychotic prescribing for patients with schizophrenia at the four hospitals

	High secure hospital	Medium secure hospitals			Low secure hospital	
		1	2	Combined		Total
Regular first-generation antipsychotic only, *n* (%)						
Oral	6 (4)	2 (4)	0 (0)	2 (2)	2 (5)	10 (4)
Depot	17 (12)	7 (13)	8 (24)	15 (17)	9 (22)	41 (15)

Regular second-generation antipsychotic only,^a^ *n* (%)						
Oral	59 (42)	8 (15)	10 (30)	18 (21)	11 (27)	88 (33)
Depot	1 (1)	9 (17)	0 (0)	9 (10)	6 (15)	16 (6)

Clozapine only, *n* (%)	33 (24)	18 (34)	10 (30)	28 (33)	8 (20)	69 (26)

Total, *n*	139	53	33	86	40	265

a. Excluding clozapine.

Clozapine was prescribed to 26% of all patients, with the highest prescription rate observed in medium security hospitals (33%).

Excluding polypharmacy and clozapine use, more patients were prescribed a second-generation oral agent than a first-generation oral agent (33% *v.* 4%); this was true for all four hospitals. In general, this pattern was reversed for depot agents, with more patients being prescribed a first-generation depot agent than a second-generation depot agent (15% *v.* 6%). 70% of patients with schizophrenia in high security were prescribed an oral antipsychotic only (including clozapine), compared with 56% of patients in medium security and 52% of patients in low security (Table 2). It emerged that 13% of patients with schizophrenia in high security were prescribed a depot antipsychotic only, compared with 28% of patients in medium security and 37% of patients in low security. Owing to the relatively small sample sizes, data from the two medium secure hospitals and one low secure hospital were combined for statistical analysis, as shown in Table 3.

**Table 3:** Oral and depot antipsychotic prescribing for schizophrenia in high security and the other hospitals

	High secure hospital	Other hospitals^a^	Total, *n*
One type of regular oral antipsychotic only, *n* (%)	98 (70)	69 (55)	167

One type of regular depot antipsychotic only, *n* (%)	18 (13)	39 (31)	57

Other,^b^ *n* (%)	23 (17)	18 (14)	41

Total, *n*	139	126	265

a. Medium secure hospital 1, medium secure hospital 2, low secure hospital.

b. More than one type of antipsychotic prescribed regularly, no regular antipsychotic prescribed.

Chi-square testing revealed a significant difference in the rate of prescribing of oral and depot antipsychotic medication between the high secure hospital and the other hospitals (χ^2^ = 12.78, d.f. = 2, *P* < 0.01). The data suggest that more oral medication was used in high security and more depot medication was used in the other hospitals. Table 4 shows the route of medication administration for patients with schizophrenia broken down by ethnicity. When medication was prescribed (i.e. excluding the ‘no antipsychotic prescribed’ category), chi-square analysis showed a statistically significant difference between the ethnic groups (χ^2^ = 6.90, d.f. = 2, *P* < 0.05); depot antipsychotics appear to be used more frequently for patients from BME groups.

**Table 4:** Medication administration for patients with schizophrenia by ethnicity

	Regular depot antipsychotic only	Regular oral antipsychotic only	Regular depot and oral antipsychotic	No antipsychotic	Total
BME patients, *n* (%)	23 (29.5)	48 (61.5)	5 (6.4)	2 (2.6)	78

White patients, *n* (%)	34 (18.2)	142 (75.9)	5 (2.7)	6 (3.2)	187

Total	57	190	10	8	265

### Consultant questionnaires

The questionnaire was sent to the 34 consultant forensic psychiatrists based at the four secure hospitals in the trust (19 at the high secure hospital, 10 at the two medium secure hospitals and 5 at the low secure hospital), as well as to the 3 forensic consultants working in community forensic services within the same trust. The overall response rate was 78% (74% high secure, 80% medium secure, 80% low secure and 100% community). Limitations in the data collected leave us unable to comment on any differences (e.g. gender, age, years of experience) between consultants who did and did not respond.

Of the hospital-based consultants responding to the questionnaire, 35% expressed a preference for oral medication and 42% expressed a preference for depot medication; 23% did not express a preference. The mean score on the 0–100 scale, where 0 indicated a preference for oral and 100 for depot medication, was 37 (s.d. = 20) for respondents from high security and 74 (s.d. = 22) for respondents from other settings (medium security and low security); ANOVA demonstrated a significant difference between the two groups (*F*_(1,24)_ = 19.759, *P* < 0.01). Thus, those working in high security preferred oral medications and those working in other settings preferred depot medications.

Most (89%) expressing a preference for oral medications worked in high security. The following reasons were given: convenience for patient, adherence, safety, less invasive, improved engagement, increased patient responsibility and improved therapeutic relationship. Most (73%) expressing a preference for depot medications worked in medium or low security, and their reasons were: adherence, clinical outcome, expectation from next level of security, reduced side-effects, reduced tension between patient and team, easier risk management in community, ‘mental health review tribunal’/‘Ministry of Justice’ reassurance, and reduced adverse events.

All of the community-based forensic consultants expressed a preference for depot medication; stated reasons included adherence, clinical outcome and convenience for the patient.

Community-based forensic consultants were asked two further questions (see Method). It emerged that route of administration was important for consultants when considering whether or not to accept a patient (mean rating for question 1, where 0 was ‘very important’ and 100 was ‘not at all important’, was 31 (s.d. = 17)). Considering question 2, consultants were also likely to accept patients currently prescribed an oral antipsychotic (not clozapine) (mean rating 72 (s.d. = 21), where 0 – very unlikely, 100 – very likely).

## Discussion

### Findings

This study demonstrates a similar rate of antipsychotic prescribing (97%) as a previous study;^6^ 3% of patients were not prescribed antipsychotic medication. Consultant psychiatrists may opt not to prescribe antipsychotic medication in the context of a drug-free trial related to diagnostic uncertainty or severe side-effects, or because a patient has refused to take such medication.

This study reveals significant differences in the antipsychotic prescribing practices of consultants working in different levels of secure care. Consultants in high security were found to prescribe more oral antipsychotics, and those in medium and low security were found to prescribe more depot antipsychotics. It may be that the likelihood of high secure patients having an extended period of supervised care ahead of them reduces the bearing of adherence on antipsychotic selection.

The overall rate of depot antipsychotic prescribing was lower than that found by Paton^6^ and also lower than that quoted in the Maudsley guidelines.^9^ Polypharmacy was less prevalent than in Paton's study;^6^ this finding was welcome but perhaps unsurprising as over a decade later the risks associated with polypharmacy are better understood and many trusts have guidelines restricting polypharmacy. The Care Quality Commission also actively discourages polypharmacy. The most common combination of clozapine augmented by a second-generation oral antipsychotic is in keeping with usual approaches to treatment-resistant schizophrenia.

BME patients with schizophrenia were significantly more likely than their White counterparts to be prescribed a depot antipsychotic. This finding builds upon existing research.^10,11^

Significant differences in the opinions expressed by consultants were found: consultants working in high security preferred oral antipsychotics and consultants working in other settings preferred depot antipsychotics. Overall, 31% of all respondents expressed a preference for oral antipsychotics and 89% of these worked in high security, whereas 48% of respondents expressed a preference for depot antipsychotics and 79% of these worked in medium and low security and the community. Comments from community consultants suggest there is no expectation that patients should be prescribed a depot antipsychotic before they are deemed suitable to be managed by community services.

It is noteworthy that the presence of a community forensic team may mean that the area served by the NHS trust in this study is not typical of other areas in England and Wales. Community forensic services are not available countrywide and it may be that general adult psychiatrists accepting patients from secure services hold different views than their forensic colleagues.

### Adherence

Both consultants preferring oral antipsychotics and those preferring depot antipsychotics listed ‘adherence’ as a reason for their preference. For patients with schizophrenia, poor adherence can be related to forgetfulness, disorganisation, complexity of regime, cost, lack of insight, ambivalence, poor relationship with therapist, stigma, side-effects and lack of perceived efficacy.^3,12^ Higher rates of non-adherence have been reported in patients with schizophrenia prescribed oral medication than those prescribed depot medication^13^ and patients treated with depot medication have been found more likely to continue medication, and to continue it for longer, than patients treated with oral medication. It has been suggested that improved adherence is likely to lead to better clinical and functional outcomes.^14^

Stone & Niz^15^ found that non-adherent patients with schizophrenia were more likely to enter the criminal justice system and suggest that consideration be given to using depot antipsychotics (or clozapine) as a first-line treatment for offenders with schizophrenia. Arango *et al*^16^ studied patients with schizophrenia and a history of violence. Of those who were violent again, those prescribed oral antipsychotics were violent sooner, and more frequently, than those prescribed depot antipsychotics. The authors link improved adherence to reductions in violence. It may therefore appear counterintuitive that the forensic population studied were prescribed less depot medication than patients in an earlier, mixed population, study^6^ and the rates quoted in the Maudsley guidelines.^9^

### Efficacy

Reviews comparing the efficacy of oral and depot antipsychotics^12,17^ report that mirror-image and some large cohort studies have favoured depot preparations but randomised controlled trials (RCTs) have not.

Lafeuille *et al*^18^ compared outcomes in patients who relapsed on an oral medication and were then ‘switched’ to a depot antipsychotic with those who remained on an oral medication. ‘Switched’ patients had fewer readmissions and fewer emergency presentations. Johnson^19^ found that 33 months after being discharged from hospital, 40% of patients prescribed depot antipsychotics relapsed, in comparison with 60% of patients prescribed oral antipsychotics. David & Adams’ review^3^ of non-forensic patients with schizophrenia identified little difference between oral and depot antipsychotics in terms of relapse rates or side-effects, but depot formulations were found superior in bringing about ‘important global change’. Leucht *et al*^20^ present a systematic review and meta-analysis of 10 RCTs carried out between 1975 and 2010; there were significantly fewer relapses in out-patients prescribed depot medication than in those prescribed oral medication.

### Psychiatrist attitude

Haddad *et al*^21^ report that 50% of psychiatrists said that their use of depot antipsychotics had reduced in the previous 5 years, and 23% said that their use had increased (the 5-year time-span included the introduction of second-generation depot agents). Despite 89% opining that depot administration was associated with better adherence, and 98% opining that depot administration was associated with reduced relapse rates, only 4% said that depot was their ‘first choice’ route of administration for patients requiring long-term treatment. The findings from the current study may go some way in explaining the possible cognitive dissonance demonstrated by Haddad *et al*'s findings, i.e. consultant psychiatrists may consider many things other than adherence when deciding on antipsychotic treatment.

Potkin *et al*^22^ reviewed prescriber-patient conversations and found that depot antipsychotics were discussed only half of the time when a patient prescribed an oral antipsychotic wished to discuss a change in medication.

### Advantages and disadvantages of depot antipsychotic medication

Previous studies have commented on perceived advantages and disadvantages of depot medications.^1,3,12–14,19,22,23^ Advantages have included a more constant plasma level, improved bioavailability, reduced availability of medication for overdose, improved adherence, more time to intervene when non-adherence is identified, reduction in family conflict and reduced treatment costs. Studies assessing patient attitude towards depot medication have revealed generally positive attitudes.^3,22^

Disadvantages have included concern about side-effects, effect on therapeutic relationship, risk of high-dose prescribing, inappropriateness of use post-neuroleptic malignant syndrome, less flexibility and delayed response (i.e. mental state improvement on initiation, side-effect reduction on discontinuation). Potkin *et al*^22^ found that the usual reason for a patient declining depot medication was needle phobia.

This study adds to the existing literature by providing a forensic perspective on the advantages and disadvantages of depot medication.

### Limitations

This study encountered a number of limitations which restrict the generalisability of the findings to wider forensic and indeed non-forensic settings. These include the small sample size, particularly with regard to the community consultants, the use of a non-validated questionnaire, and not including patients in the private sector (who in 2007 accounted for 35% of patients in England).^24^

### Clinical implications

Community teams caring for forensic patients with schizophrenia do not seem to have an expectation that patients should be prescribed a depot antipsychotic medication before they are deemed suitable for their service. It could be argued that given the relatively low rate of depot antipsychotic prescribing found in this study, and the superiority of depot preparations in terms of adherence, readmission and relapse rates, ‘important global change’ and, notably, risk of violence revealed through this literature review, consultants working with forensic populations should consider taking steps to increase their use of depot antipsychotic medications.

### Directions for future research

Future research could attempt to evaluate the opinions of a more representative sample of consultant forensic psychiatrists, or the opinions of general adult psychiatrists and non-medical practitioners, particularly nurses,^3^ who may be expected to receive the handover of patients formerly known to forensic services. It would also be interesting to establish whether or not the perceived effectiveness of different types of antipsychotics influences prescribers' choices. Future research could further explore the finding that BME patients are more likely to be prescribed a depot antipsychotic than their White counterparts. It may also be useful to undertake a follow-up study, where patients stepped down from high or medium security are followed up some time after discharge and any changes in antipsychotic prescription are identified and investigated.
